# Re-thinking breast and cervical cancer preventive campaigns in developing countries: the case for interventions at high schools

**DOI:** 10.1186/s12889-019-6890-2

**Published:** 2019-05-03

**Authors:** Chris Onyebuchi Ifediora

**Affiliations:** 1 Onyebuchi Chris Ifediora (OCI) Foundation, Suite 2a, Cyfed Complex, Ifite-Amansea Road (Opposite UNIZIK Permanent Site Gate), Awka, Nigeria; 2 Griffith University Medical School, Gold Coast, Australia

**Keywords:** Breast, Cervix, Cancer, Women, Intervention, Prevention, High school, Teenager

## Abstract

**Background:**

The negative impact of cervical and breast cancers in low and lower-middle income countries are worsening, and, along with other non-communicable diseases, occur disproportionately in these resource-limited economies. Most preventive approaches to these cancers require government funding, but few countries with the most at-risk population can afford government-sponsored universal vaccination, screening, diagnostic and treatment programmes, which, along with socioeconomic issues, contribute to the poor outcomes in these mostly developing countries. An urgent need exists, therefore, to find an effective, affordable, cost-effective, culturally-acceptable and sustainable way of reducing these cancers. This paper advocates a re-thinking in the current preventive campaigns.

**Main body:**

Using evidence provided by recently-published papers, a case is made for enlightenment campaigns to primarily target teenagers (boys and girls) in high schools of developing countries. Inclusions into the schools’ academic curricula are the recommended approach, given that both cancers take hold on populations within that age bracket.

This approach, if adopted, may be the only accessible, affordable and realistic approach that gives millions of women in low and lower-middle income countries the chance at survival. Empowering them early instils the self-awareness and confidence necessary for young adults to take charge of their own health. The acquired knowledge, in turn, helps them adopt positive attitudes and preventive behaviours that will, ultimately, prolong their lives.

**Conclusion:**

The recommended approach offers governments and concerned stakeholders an evidence-based option that allows them to deliver cost-effective and sustainable life-saving interventions, while hoping to get around the bottlenecks that limit the large scale implementation of other effective but capital-intensive strategies.

**Electronic supplementary material:**

The online version of this article (10.1186/s12889-019-6890-2) contains supplementary material, which is available to authorized users.

## Background

Estimates suggest that, excluding expenditures on diagnosis and treatment, the global costs from premature deaths related to non-communicable diseases might exceed $US30 trillion in the next 20 years [1]. Currently, about 40 million deaths per year are attributable to these diseases, with 31 million (78%) of them coming from low and lower-middle income countries [1]. Ironically, just 1% of universal health funding is earmarked for preventing them [1], with current attention in the developing world focused on communicable, non-cancer, infective diseases like HIV, malaria and tuberculosis [2], as well as childhood immunizations [3].

Two major non-communicable diseases with significant impact in the developing world (which largely make up these low to lower-middle income countries), are cervical and breast cancers [4]. Available data reveal that, even though cervical cancer is the fourth commonest cancer in women worldwide [5], it remains the second most common in developing countries, particularly among women aged between 15 and 44 years [6]. Data from a few years ago reveal that an estimated 445,000 new cases, which represented 84% of all global new cervical cancers, were reported in developing countries [5]. Also, of about 270,000 global deaths from the cancer in that same period, over 85% occurred in these same countries [5].

On its part, breast cancer accounts for 11.6% of all cancers and is the commonest malignancy worldwide (apart from lung and non-melanoma skin cancers) [7]. It is the fifth most common cause of cancer deaths worldwide, accounting for 571,000 deaths in 2015 [8]. Its incidence has risen steadily from 1-in-20 women in 1960, to the current 1-in-8 [9]. In recent years, increased rates are being reported from developing countries, where incidences are gradually matching levels hitherto seen only in high-income countries, possibly because of the increased adoption of western lifestyles [10].

Of note is that the widespread, mostly government-funded universal vaccination, screening, diagnostic and treatment programmes open to women in high-income countries are not available to those in disadvantaged, developing economies, despite the disproportionate burdens face by the latter group [5]. In view of this, the need arises to find an effective, affordable, cost-effective, culturally-acceptable and sustainable way of reducing the duo of cervical and breast cancers in these areas. Using evidence from recent and existing research publications in Nigeria, a quintessential lower-middle income country, this paper argues that a re-thinking in the current preventive campaign strategies is needed if the current negative trend is to be curtailed [4].

A case will be made for these campaigns to primarily target teenage youths (girls and boys) in developing countries. This is necessary given that, cervical cancer particularly, and breast cancer to a reasonable extent, take hold at young ages. This paper will argue that integrating preventive cancer strategies into the academic curricula of senior secondary (high) school students (who are mainly in their mid-teens) in these developing countries is one area stakeholders need to pay attention to.

## Main text

### Cancer burdens in developing countries: insights from Nigeria

The aforestated approach, if adopted, will arguably save hundreds of thousands, if not millions, of lives, in low and lower-middle income countries. It also presents an evidence-based option that will help complement existing policies on the cancers, by providing realistic and implementable activities. Even though this paper’s viewpoints are applicable to developing countries with disproportionate breast and cervical cancer burdens, the primary focus is on Nigeria. This focus is warranted by the fact that the key publications that informed the thinking expressed in this paper were all from studies carried out in Nigeria. In addition, with 54% of Nigeria’s estimated 200 million population in 2018 being women, the disease burden from the country, is huge [11]. Available data, cited below, reveal disturbing trends faced by Nigerian women regarding breast and cervical cancers, and provides further justification for the focus on this country.

Nigeria is a country located in West Africa, and is categorized as a lower-middle income country [4]. About 50.3 million of the country’s women aged 15 years or more are at risk of developing cervical cancer, and in 2017, a total of 8240 of the 14,089 Nigerian women diagnosed with the cancer, died [6, 12]. Equally worrying is the projection that, by the year 2025, cervical cancer deaths among Nigerian women would rise by 63 and 50% respectively for those aged “≤ 65” and “> 65” years [13]. These disturbing figures may also applicable to other developing regions of the world, particularly when compared to the developed ones. For instance, a 2018 report from the Global Cancer Incidence, Mortality and Prevalence (GLOBOCAN) data reveal that the age-standardized rates (ASRs) on incidence and mortality from cervical cancers (per 100,000 women per year) in North America were respectively 6.4 and 1.9 respectively, while in Western Europe, they were 6.8 and 2.1 [7]. These numbers are much less than those from the less developed regions of the world like Western Africa (29.6 and 23.0 respectively for incidence and mortality), Eastern Africa (40.1 and 30.0) and South-eastern Asia (17.2 and 10.0) [7]. Despite these disappointing numbers, in Africa, a continent that includes Nigeria and other developing countries, only nine of 55 countries have functional cervical cancer preventive programs [14].

For breast cancer, despite the much higher age-standardized incidence rates (per 100,000 women per year) reported among developed countries in Western Europe (92.6) and North America (84.8), their mortality rates were respectively low at 15.5 and 12.6 [7]. In contrast, even though breast cancer incidences were relatively low in the less developed areas of the world (37.3 in West Africa and 38.1 in South-eastern Asia), the mortality rates were much higher (17.8 in West Africa and 14.1 in SE Asia) relative to the already-cited figures from the more developed world [7]. Sadly, specific burdens in individual developing countries like Nigeria are not well known due to poor and varying records [15], but the 10% five-year survival rates reported by a Nigerian study compares poorly to the 70% survival rates from high-income countries in Western Europe and North America [15, 16].

The numbers above provide compelling reasons for actions against breast and cervical cancers in Nigeria and other developing countries, and further confirms the already-known fact that the existing policies in these areas are not working (by not being implemented). As already stated, this paper advocates a different thinking that can help reduce this burden if adopted and implemented in developing countries.

### Current preventive measures and realities from developing countries

#### Cervical cancer

The preventive techniques are anchored on the fact that nearly all cases of cervical cancer are attributable to the Human Papilloma Virus (HPV), a sexually transmissible virus with most infections occurring soon after the very first sexual activity [5, 17, 18]. The fights against cervical cancer usually involve the trio of lifestyle modifications, vaccinations and screenings.

Lifestyle modifications aim to limit exposures to the HPV through the delay in the age of first sexual intercourse, as well as avoiding multiple sexual partners, unprotected pre-marital sex, tobacco smoking, and having more than four babies, among others [5]. Even though all these measures are either free or of low-cost, a recent survey revealed that many high school girls in Nigeria are unaware of them [19].

The use of vaccines presents another preventive measure against HPV. *Gardasil* and *Cervarix* have both been available in global markets since 2007–2009 for this purpose, and are recommended by the WHO for boys and girls aged 9 to 13 years [13]. Unfortunately, of the estimated 118 million women targeted worldwide through these programmes in 2014, only 1% were from low to lower-middle income countries [20], and, as at 2016, only 65 of over 120 countries that approved the vaccines had government-sponsored programs, and most are of high-income economies [5].

The final major preventive approach to cervical cancer is through screenings to detect early lesions (using Pap Test, Liquid-based cytology, or HPV testing) [21]. Just like the vaccines, this approach is beyond the reach of most at-risk women in developing countries, apparently due to costs. This reality is revealed by estimates from the WHO, which shows that in a country like Nigeria, where the HPV prevalence was 16.0% as at 2017, the coverage of cervical cancer screening by age (in years) were 1.8% for those aged 25–34, 6.6% for those 35–44, 12.7% for those 45–54, and 2.8% for those 55–64 [6]. Statistics from other developing countries were not available for comparison, but the coverage levels are expected to be as poor, given that only nine of 55 African countries have national anti-cervical cancer programs in place [14]. These numbers are very disappointing, and stand in contrast to figures from developed countries, where a country like Sweden reports up to 90% screening uptake [22]. Unfortunately, cervical cancers and HPV infections are asymptomatic, and most screening tests in Nigeria are opportunistic, meaning that most affected women present too late to be saved [23].

#### Breast cancer

Two key facilitators for reducing breast cancer deaths are “health education” and “breast screening” [24]. Health education basically empowers women to be “breast aware”, teaching them the risk factors and early symptoms of breast cancers, as well as the techniques to detect these symptoms.

For breast screening, the main available method is “breast imaging” through ultrasonography and/or mammography. Even though not exactly screening techniques, breast self-examination (BSE) and clinical breast examination (CBE) also help in identifying suspicious lesions [25]. Unfortunately, the wide-scale utilization of CBE and breast imaging in resource-limited developing countries are still poor due to significant financial and manpower limitations [26]. In contrast, BSE offers a simple, cheap, and non-invasive technique that can be practised by virtually anyone [15, 24, 27], and is recommended monthly from the age of 20 years [9]. Studies have revealed that BSEs alone do not lead to reductions in the rates of breast cancers [28], but, if combined with imaging and CBE, there is evidence that they can improve outcomes and reduce mortality by as much as 25% [29]. Beyond this, regular BSE practice offers further advantages because, by improving body awareness, it allows changes potentially indicative of breast cancer to be picked up early [30, 31], ultimately facilitating the use of efficient and less aggressive treatment modalities [27, 32]. Give these positives, it comes as little surprise that the *Royal College of Nursing of the United Kingdom* actively encourages the promotion of breast awareness, of which BSE is a key component of [28].

Generally, African women affected by breast cancer present at least a decade earlier than their Caucasian counterparts [33]. In Nigeria, up to 69% of breast cancer cases occur among pre-menopausal women aged 26 to 50 years, with as many as 64% presenting with advanced diseases [34]. Only 7% of affected Nigerian women present within one month of symptom discovery [34], while 70% are seen after a prolonged delay of at least three months, contrasting with the less than 30% of Caucasian women that delays this much [35]. Given the relatively young age and the late presentation, most lesions in African women are bigger, more aggressive, advanced, and with poor prospects of long-term survivals, Therefore, any intervention to make their women “breast-aware” at an early age, should be encouraged [36].

Unfortunately, despite the foregoing, recent studies reveal that young women in developing countries are not breast-aware, with low levels of knowledge on the specific risk factors and early symptoms of breast cancer, as well as the BSE techniques (timing, frequency, and technique) [24, 37, 38]. Furthermore, the actual practices of BSE stood at 6.1% [37], 10.1% [24] and 15.5% [38] across high schools in Nigeria. While these numbers may not be completely worse than those from some high or upper-middle income countries like Turkey [39] and Saudi Arabia [30], for most women in Nigeria and other low or lower-middle income countries, being breast-aware may be their only chance against the cancer, and efforts should, therefore, be made to improve on these numbers. This paper advocates a new, effective and all-encompassing way of doing this.

### New THINKINGS against the cancers: the evidence

The analyses in the preceding paragraphs raise the question of how women in these disadvantaged countries, who have little access to reliable screening and treatment methods despite bearing the major brunt of breast and cervical cancers, can be effectively empowered to protect themselves.

The identified differences in cancer data between developed and developing countries are not solely due to socioeconomic differences. They have, in fact, been attributed largely to the poor screening programs and awareness among women in the latter group of countries [40, 41]. Worse still, early treatments are rare in these countries, resulting in poor outcomes and high mortality rates [33]. This contrasts facts from developed countries, where up to 80% of cervical cancers are managed early [5].

While there are existing policies by governments in developing countries to prevent, screen, and treat these cancers, a significant policy-implementation gap limits their efficacy [1]. As a matter of fact, most of these countries lack organised, government-sponsored preventive programs [5, 40]. .Ironically, even though the cost of HPV vaccinations in poor countries can be as low as US$ 4.50 per vaccine (thanks to the support from the Gavi Alliance), significant set-up costs are still needed since the basic infrastructure required to ensure proper service-delivery (like recall and follow-up systems) are virtually non-existent in these areas [42]. These costs partly limit the policy implementations. Unfortunately, this trend is likely to remain, given that global health funding disproportionately favours communicable diseases ahead of non-communicable ones like cervical and breast cancers [1].

How long it will take for developing countries to catch up with their counterparts in the developed world, with respect to tackling these cancers, may be up for debate, but what is not debatable is that, for the population at risk in these countries, time is slipping irreversibly away. Evidence exists that raising awareness to the existence of these cancers and their preventive strategies (lifestyle practices, screenings, and vaccinations) can help reduce their incidence, morbidity, and mortality rates [13, 43]. Therefore, empowering women in these respects is an option already embraced by many enlightenment campaigns, but this paper argues that a more effective approach is to start from the high school years, when most women are teenagers. This measure will not only be timely, but, if integrated into the school curricular of senior secondary schools in culturally acceptable ways, will be cost-effective and sustainable. It is also recommended that boys be included in the campaign, given that sexual pressure from men contributes significantly to the problems. They should, therefore, be part of the solution, if long term beneficial outcomes are to be realized.

It is also very important to emphasize that the approach being advocated for in this paper is meant to complement, not replace, the proven preventive and screening systems already in place for breast and cervical cancers. However, the unfortunate reality is that, for the foreseeable future at least, they might remain the only options available to hundreds of millions of women in many developing countries. Even where they are not the only options, they will still provide useful adjuncts to any established programs, given the expert recommendation that awareness campaigns need to form key components of any national screening and vaccination programs in developing countries if they are to be effective and sustainable [42]. This recommendation is on the basis that, to be successful, national programs rely on a number of key factors, like functional recall systems, reliable registry of women and their screening histories, appropriate follow-up and treatment systems for positive results, as well as quality control measures [42]. These are all lacking in most developing counties like Nigeria. As such, directly empowering women would strengthen any systemic weaknesses in the health infrastructures in these areas, and allow them to actively seek for the interventions. This argument further underlines the need to take the evidence-based suggestions of this paper seriously, as it not only complements any national programs that governments and concerned stakeholders might have, but also offers outright alternatives for implementing affordable and life-saving measures in places where no programs exist.

### Justification for the high school approach

About 72.8% of Nigerians aged 15 to 24 years are literate [44], so any school-based program will reach a majority of young adults in the country. The supporting pieces of evidence in support of the views expressed in this paper, available from peer-reviewed publications, are now discussed in the ensuing paragraphs.

#### Cervical cancers

Firstly, most cervical cancer cases in developing countries present among women in their mid-30s, an age which is up to 15 years earlier than occurrences among women in developed countries [33]. Given that most HPV infections take 10 to 20 years before progressing to full-blown cervical cancers [45], a reasonable assumption is that the earliest exposures to clinically significant HPV infections would be in the mid-teens, before or shortly after the age of 20 years [33]. This is consistent with the report that the age of first sexual exposure for women in Nigeria is 16.7 to 17.9 years [46], which, incidentally, is the age at which most females are in the final year of their high schools [19]. In addition, it has been reported that up to 12% of cervical cancer cases in developing countries occur in women under 30 years of age [47].

Another reason for advocating anti-cervical cancer interventions in high schools is based on the fact that the proportion of sexually active females in Nigerian tertiary institutions (like universities) range from 71.2% [48] to 81.5% [33]. This means that, for a good proportion of women in Nigerian tertiary institutions (usually in their late teens to mid-twenties), potential exposures to HPV would have already occurred. Notably, only 15.6% of 16-year-old Nigerian women (likely to be in high schools) are sexually active, while as many as 51.7% were already sexually active before the age of 20 years [6]. Unfortunately, going by the results of a recent survey, most of these women would be unaware of the associations between sexual exposure and cervical cancer [19]. The survey, completed in 2017, explored the knowledge and attitudes of 321 female senior secondary school students in South-eastern Nigeria (mean age 16.8 +/− 1.5 years), and found that only 22% of them had heard of HPV, while less than a third knew that it could be contracted through sexual intercourse [19]. The study also found that those who had heard about cervical cancers were statistically more likely to be aware of a number of its risk factors and early warning symptoms.

#### Breast cancers

Regarding breast cancers, it is known that the cumulative frequencies of occurrences were 0.8% at age ≤ 20 years and 3.3% at < 25 years [49]. In fact, a case has been reported in a patient as young as 14 years [49]. Despite this risk faced by young adults, a 2017 study of teenage females in Nigerian high school students observed a poor level of specific knowledge on breast cancer risk factors and its early warning symptoms, and noted that only 6.1% of them practised monthly BSE, with very few knowing the correct techniques and timing [37]. Given that prognosis in younger patients are poor [50], early screening and detection among the younger age group are vital in developing countries, where no other functional preventive programs are in place [30]. Such early detection is linked to a reduction in associated morbidities and mortality [26], as prognosis has a direct association with the tumour localization and stage at diagnosis [9].

A final reason for advocating interventions in high schools, which is applicable to both cervical and breast cancers, is that, most teenage participants will, at some point in their lives, attend higher institutions, become mothers, get employed, and get involved in wider community activities. They, therefore, have many years and opportunities to pass on the knowledge to others, including their children, grandchildren, friends, school mates, work colleagues, and others in the society. The impact of empowering them early will, therefore, be multiplicative, and has a long-term spill-over effect in the societies concerned.

In view of the foregoing discussions, it becomes obvious that the duo of cervical and breast cancers have their roots in early adulthood, and delaying enlightenment interventions to mid or late adulthood would come late to many, as exposure to irreversible risks might have occurred. This problem is of a huge significance in most developing countries like Nigeria, which have little to no organized, all-encompassing, government-sponsored preventive programs. For clarity, most of these countries have good policies, but, as recently acknowledged in a WHO publication, a significant policy-implementation gap exists, and alternatives need to be found if outcomes will match the compelling needs for action [1].

One rather worrying fact is that that there exists no reliable source of information on these cancers for high school students in Nigeria, with recent publications revealing poor levels across all sources, including schools [19, 37]. Enquiries reveal that the current curriculum for schools in Nigeria does not contain teachings on these cancers, and consultations with technocrats involved in high school curriculum design and implementation suggest that the necessary curriculum change is feasible. Given the modest positive attitudes by young females in Nigerian schools [19, 37], such changes will be welcomed, and would, therefore, most likely be effective. Unfortunately, even though the electronic and print media, as well as the internet, are good sources of the required information in the developed world, they are not readily accessible to most Nigerians, given the poor power supply, internet coverage, and relatively unaffordable costs.

To ensure a sustained efficacy of the interventions over the medium to long-term, available evidence from recent longitudinal studies indicate that the advocated curriculum change should incorporate in-built evaluation systems (like examinations and quizzes) as a way of ensuring “engagement” [51, 52]. These six-month longitudinal studies observed that, participants who were “engaged” with breast and cervical cancer awareness campaigns (either by reading relevant handouts or by attending empowerment symposium) showed significant improvement in knowledge and practice of monthly BSEs. These improvements were not observed among the “unengaged” participants.

To further increase the chances of sustaining the gained knowledge over time, yearly repetition of the same items across the final three classes in Nigerian high schools for each student cohort will be important. This recommendation is based on the fact that ‘repetition’ is a proven way of transforming learned behaviours into habits, and will, therefore, be vital if the teachings recommended in this paper are to become long term habits for the participants [53, 54].

It is worth pointing out that in countries or regions where other health initiatives are already part of the school curriculum, the suggested initiatives of this paper can be integrated into such existing programs. For instance, in Nigeria, there is the “National Family Life and HIV Education”, which allowed the 2003 introduction of anti-HIV contents into the curriculum of junior secondary school students in some parts of Nigeria [55]. The fact that a program of that nature is in existence strengthens the case that the current initiative is feasible, and sponsoring organizations can build from the templates provided by that. It should be noted, though, that the currently proposed initiative is unique in a number of ways. Firstly, this paper targets senior (not junior) secondary school students. Secondly, this paper advocates measurable and trackable assessment systems that will allow evaluation, with repetitions across three different classes each student cohort. Thirdly, the ideas expressed in this paper are original and informed by empirical evidence primarily developed from the evaluation of recent interventions in Nigeria. Finally, as shown in a tentative implementation plan (Additional file 1), this recommendations of this paper integrates technology that will improve its chances of success, as a key component of its implementation will be the availability of online, electronic support system, along with downloadable mobile phone application to support engagement and sustainability wherever possible.

## Conclusions

In summary, this paper has presented the novel argument that introducing cervical and breast cancer prevention strategies into the academic curricula of high schools in order to target teenage women is necessary, and will be timely, sustainable, and effective. It will also reach out to a large number of women at minimal costs to individuals, families, governments, and private organizations. The need to embark on this project is pressing, given that, for tens of millions of women in low and lower-middle income countries, the suggested approach may be all that gives them the chance at survival against these deadly cancers.

The suggestions in this paper are very feasible, as is shown in a modelled implementation plan for Nigeria. This is shown in Additional file 1, and shows specific aims and success measures, a step-by-step plan, and a list of potential stakeholders to be involved in a tentative project.

### Potential limitations, implications and solutions

A number of barriers to the actualization of the proposed recommendation have been identified. For the proposals of this paper to be successful, these potential limitations need to be surmounted. They include:

1. There may be cultural and religious resistances to the recommendations, given that some of the societies involved are very conservative, and may perceive the strategies as incompatible to their beliefs. To overcome this, the early engagement of key stakeholders is important (Please see the “Proposed Implementation Committee” in Additional file 1*).* Through this, the harmlessness of the intervention can be highlighted. It also creates room for explaining the danger of doing nothing, while acceptable, socially and culturally acceptable formats for delivering the messages can be agreed on. In addition, obtaining formal ethical approvals from the relevant authorities will help in assuaging the concerns as they arise.
2. The initial implementation costs may also pose a problem, given that thousands of education materials may need to be provided for all the participating high schools, while hundreds of teachers may need to be trained to deliver the required activities. While theoretically, this can be a challenge, partnerships with governmental and non-governmental organizations will minimize these costs. In addition, engaging already-employed teachers in Nigerian high schools will help circumvent the need for recruiting new teachers. Combined, these measures will ultimately lead to the implementation of a high-impact and low-cost project.
3. The long-term sustenance of the program might also be a challenge, given the need to sustain interest, ensure engagement, and not deviate from the aims. To overcome this, there will be a need to regularly evaluate the measures, so as to ensure that the aims will constantly be met. Examinations and quizzes in high schools will also help in overcoming this potential challenge, along with the adoption of technology (See Additional file 1).

## Additional file

Additional file 1: Suggested implementation plan and roll-out approach for introducing anti-breast and cervical teachings into the curriculum of Nigerian high schools. This material presents a proposed intervention template for the recommendations of this paper. It covers the aims and objectives, as well as an outline of a potential step-by-step implementation and the composition of a likely stakeholder committee. (DOCX 23 kb)

## Abbreviations

ASR: Age-standardized rates
BSE: Breast self-examination
CBE: Clinical breast examination
GLOBOCAN: Global Cancer Incidence, Mortality and Prevalence
HIV: Human immune-deficiency syndrome
HPV: Human Papilloma Virus
NGO: Non Governmental Organisation
OCI: Onyebuchi Chris Ifediora
PPSSC: Post Primary Schools Service Commission
WHO: World Health Organisation
