# Supplementary material for: Re-thinking breast and cervical cancer preventive campaigns in developing countries: the case for interventions at high schools
Source: BMC Public Health. 2019 May 3;19:503. doi: 10.1186/s12889-019-6890-2 (PMC6500011; doi:10.1186/s12889-019-6890-2)
Supplement: Supplementary file 1 — Suggested implementation plan and roll-out approach for introducing anti-breast and cervical teachings into the curriculum of Nigerian high schools. This material presents a proposed intervention template for the recommendations of this paper. It covers the aims and objectives, as well as an outline of a potential step-by-step implementation and the composition of a likely stakeholder committee. (DOCX 23 kb) [file 12889_2019_6890_MOESM1_ESM.docx]

**Additional file 1**

SUGGESTED IMPLEMENTATION PLAN AND ROLL-OUT APPROACH FOR INTRODUCING ANTI-BREAST AND CERVICAL TEACHINGS INTO THE CURRICULUM OF NIGERIAN HIGH SCHOOLS

While this paper recommends that every region and country should forge their own curriculum based on their peculiarities, a proposed template, based on Nigeria, has been suggested here. The sections below cover the aims and objectives, an outline of a step-by-step implementation, and proposed stakeholder committee.

1. **AIMS AND OBJECTIVES**

To achieve engagement, the proposed interventions will be repeated for each student cohort as they go through the three classes in Nigerian High or Senior Secondary Schools (SSS Classes 1, II and III). There will also be a single end-of-year (or term) examination for each class.

The program has quantifiable objectives that can be tracked at “individual-student” and “whole-school” levels, using already validated tools used in the published studies that informed this whole approach. Data collection will be through post-lecture quizzes and end-of-term examinations. The specific objectives are:

1. After the first 12 months, ≥80% (≥90% in 24 months) of the participating students (males and females) would have satisfactory knowledge on the (1) **Risks** (2) **Symptoms** and (3) **Preventive Practices** against breast and cervical cancers;
2. Among the female participants, ≥50% would be practising monthly breast self-examinations (BSE) after the first 12 months, and ≥70% after 24 months. A mobile phone application that can send monthly reminders for BSEs will be helpful;
3. The “whole school” measure aims that ≥80% of all eligible schools in any region of intervention will be involved in the campaign within the first 12 months, with at least 90% expected after 24 months;
4. There will also be **qualitative** evaluations, to be obtained through direct feedbacks from the teachers, schools, and other stakeholders that will be involved in the project.
5. **PROPOSED STEP-BY-STEP IMPLEMENTATION PLAN**
6. A one to two-year roll out period may be necessary;
7. A phased roll-out over a 5-year period is suggested for the whole country. This can be state by state across Nigeria’s 36 states and Federal Capital territory. Some states can be done in batches depending on logistics and readiness;
8. The teachings will be introduced into the regular academic curricula of all the SSS classes. It can be done once per academic year, but will be repeated for each student cohort as they pass through SSS 1, SSS 2, and SSS3 respectively;
9. A subject compulsory for all SSS students in the region will be necessary. Civic Education in Anambra State of Nigeria is an example of such a subject, but every state or region should identify theirs;
10. A pre-intervention workshop will be held for all the teachers of the identified subject in the eligible classes. This will help prepare them well for the expected changes in the curriculum, the teaching contents and delivery methods, the examinations, and the reporting/submission of results;
11. Based on experiences in past symposium activities, a 45-minute teaching per class will suffice;
12. An immediate post-lecture quiz may be necessary, but an end-of-term (end-of-year) examination for each cohort from SSS 1 to SSS 3 will be compulsory. These will help improve engagement, and assist in data collection for tracking the intervention;
13. Each school will nominate a staff that will co-ordinate and submit the results of their quizzes and examinations to the sponsoring organization. A secured, dedicated, electronic, web-based portal for result submission should be made available, where feasible;
14. A section of that webpage should be freely accessible to everyone teachers, participants and other women), and should contain information relating to the campaign, along with support and link to video demonstrations of breast self-examinations, BSE);
15. Based on the performances in the end-of-year examinations, yearly awards for the best students, and/or schools will be important to motivate and sustain participation and excellence;
16. Books and other relevant anti-breast and cervical cancer materials should be made available to all the participating schools. These will be kept in their libraries and accessed by the students (and interested staff) on a “use-and-return” basis;
17. A free Mobile Phone Application is recommended. It should contain useful resources as the website, along with the ability to send monthly reminders to the participants (and all other women in Nigeria and beyond willing to use it). This will help increase motivation and commitment towards the BSE, and provide easy access to relevant resources;
18. Where feasible, publicity campaigns ahead of the campaign roll-outs are recommended, so as to increase buy-in from the targeted communities. This might include television appearances, advertisements, press conferences, road walks, courtesy visits (to schools and other stakeholders like government officials, traditional rulers, and so on). Flyers, posters and outfits (t-shirts, caps, badges) will also be useful;
19. The capital for the activities above are likely to be one-offs. Not all of the propositions are not compulsory for the success of the initiative, and will be optional to the sponsoring organizations and states/regions. Where available though, they will provide important complements that will improve engagement to the program, help sustain the lessons, and widen the scope of the beneficiaries. Over time, the costs will be insignificant compared to the potential gains.
20. **PROPOSED IMPLEMENTATION (WORKING) COMMITTEE**

To ensure that the program is all-inclusive, and that all viewpoints are taken on board and concerns are addressed, a working committee that will help plan and execute the programs will be necessary. The proposed committee should include:

1. Representative(s) of the main sponsoring organization (NGOs, Governments, etc.);
2. Representatives from the involved schools;
3. Public health experts that will be involved in the pre-intervention workshops;
4. At least two **Campaign Champions.** These should be influential technocrats from the health and education sectors in the states or countries where the intervention is to take place. They will be useful in navigating potentially tricky negotiations, while creating access to relevant stakeholders. They will also help in aligning the project ideas to the perculiarities of the concerned communities, and, ultimately help in tailoring the interventions to be socially and culturally acceptable;
5. Government representatives and other stakeholders in education, health, and women sectors in the concerned communities,
6. Representations from all relevant religious faiths, including the traditional institution;
7. Representtives of the parents, possibly co-opted through exsting Parent-Teachers’ associations.
